# The Week's Work

**Published:** 1909-12-25

**Authors:** 


### The Week's Work.

### THE SECRETARY AS EDITOR.

Our energetic contemporary *The Hospital Gazette*, which is the official journal of the Hospital Officers' Association, is showing commendable brightness and vigour under the direction of its new editor, Mr. Stewart Johnson. He seems to have a merry wit, and wit is far too infrequent in the present day. We have always believed in the cultivation of a cheerful countenance, especially among workers in hospitals, whose life is so largely spent in an atmosphere of suffering, and amidst scenes which might otherwise prove at times unendurable. Cheerfulness in the sick is the high road to recovery, and cheerfulness in the hospital official is the best asset that men or women working in hospitals can have, for so long as they continue cheerful they are bound to be healthy and vigorous. So we welcome the new spirit in hospital journalism, which we owe to Mr. Johnson, whom we shall expect to see rise steadily in his profession. We hope he may long continue to provide his readers with most excellent copy.

### THE HARTSHILL TRAGEDY.

It is remarkable that Mr. Bryce, the late House-Governor and Secretary of the North Staffordshire Infirmary, should have suddenly left his work without notice, and that Mr. Henry Roscoe, M.R.C.S., L.R.C.P., D.Ph., who so recently was appointed his successor, should have gone to the North Staffordshire Hotel at Stoke to take up his appointment, and there have committed suicide on Friday night. Nothing appears to have been known of the cause of the tragedy, as Mr. Roscoe, in two letters which he left behind him, merely expressed regret to the hotel manager, and left all his effects to his half-sister, Miss Jessie Roscoe. It is stated that there is some evidence to show that he had suffered from severe illness on a former occasion, and that he was at the time of his death feeling depressed and low, and might possibly have been suffering from his old complaint. These matters will probably be cleared up at the inquest. The North Staffordshire Infirmary is going through a period of reconstruction, and we heartily sympathise with the committee and authorities in the present circumstances which they have to face. In view of the proposed increase of the staff and the amendment of the rules, we hope that steps will be taken to consider carefully whether it might not be best in all the circumstances to appoint a medical superintendent rather than a house-governor and secretary to fill the vacancy caused by Mr. Roscoe's death.

### GOOD FOR THE SUNDAY FUND.

The Rev. L. S. Lewis, who is an ardent Anti-Vivisectionist, made a good point at the meeting of the Constituents of the Metropolitan Hospital Sunday Fund, on the 17th instant. Under the laws of the constitution of this fund, the contributing congregations are entitled to a voice in the management of the fund, which they can obtain by nominating their minister and two laymen to represent them at the annual meeting of the constituents, which is held at the Mansion House at the end of each year. A year ago Mr. Lewis, on attending the meeting of constituents, was ruled out of order when he rose to express the views of himself and those he represented in regard to certain matters contained in the report of the Council, which was submitted to the meeting for approval. This action on the part of those responsible Mr. Lewis ascertained from the solicitors of the fund to be illegal, and he gave notice of a motion to alter Law 3 of the Constitution, with the object of vesting the election of the Committee of Distribution in the constituents at the annual general meeting. This proposal was most objectionable, and, if carried, would have destroyed much of the value of the Metropolitan Hospital Sunday Fund. Mr. Lewis, who is a gentleman doing excellent work in a poor district, whom everybody respects, was approached. He recognised the force of these objections, and at the meeting on the 17th instant supported the adoption of the report, and withdrew his resolution. In doing this, Mr. Lewis was careful to elicit from the Lord Mayor that every representative attending the annual meeting was within his rights when he rose to discuss questions arising out of the report before its adoption. Mr. Lewis has done good service in this matter, for unless the rights of the constituents had been recognised and supported by the Council, the fund must speedily have lost its popularity, and might gradually have sunk into insignificance.

### SELF-SUPPRESSION TO SUCCESS.

The universities and colleges handicap their graduates for the battle of life by not devoting six months or a year of the college course to the enforcement of common-sense manliness. A great ambassador once told the writer that if he did not laugh at the follies of men and women he must perforce take to suicide. That was the merry side of self-suppression, and self-suppression for a young man is the high road to success, especially in the medical profession. A good deal of criticism has arisen amongst candidates this week for a vacant honorary medical post now filled. We believe, however cruel it might prove to be, that it would be kind and helpful for a reporter to attend the meeting of the Election Committee on an occasion like this, so that he might give a word-photograph of the proceedings and exhibit each candidate as he actually appeared before the Committee. It is aggravating, of course, for a young and enthusiastic surgeon to be asked whether he is qualified in all the duties of a physician, but self-suppression before laymen in such circumstances may mean all the difference between success and failure. To a lay committeeman often a resident medical post is a resident medical post and nothing more. He may know nothing, and care less, about the professional duties typified by the house appointments, or the precise significance of the title each officer may bear. So when the day of election comes, personality is the best asset a candidate may have, for the most surprising appointments are often made in consequence of the strong individuality of the successful man. Nor is this on the whole to be regretted, for the medical staff of a great hospital to be at its best must partake largely of the nature of a family circle, where the best interests of the hospital are the first consideration with every member of it. Of course, due weight should attach to qualifications and experience, but for the post of an assistant, especially where time and opportunity will be ample to test the merits and higher qualifications of the juniors for senior posts, individuality and character always have told, and always will tell, and it is well that they should do so. Every man who is ambitious should commence his fight for place by self-discipline and self-sacrifice. He thus attains a widespread reputation for what is known as the "bedside manner," and is bound ultimately to secure for himself a career and make a name which would otherwise be unattainable.

### SIR EDMUND HAY CURRIE AND HOLIDAYS.

Sir E. Hay Currie, who is over seventy years of age, shows commendable energy and zeal in the furtherance of the Hospital Sunday Fund. We were glad to note that the announcement of the fact that he is going to the South of France for a long holiday was received with sympathetic cheers by the audience in the Egyptian Hall of the Mansion House on Friday last. We have often insisted that the adequate remuneration of the men and women who devote their lives to the cause of the sick should be the especial care of the committees whose servants they are. We would go further, for we maintain that the health of these officers is a material asset in the life of every voluntary institution, and that every facility should be afforded to each zealous worker to take a good holiday, as in the case of Sir Edmund Currie. It is matter for congratulation, too, that a new spirit is spreading throughout the hospital world, which has secured the gradual and extended recognition of the principle that, on the attainment of a certain age, hospital officials who have devoted the best part of their lives to hospital work are entitled to receive an adequate pension. We are glad to have an opportunity to call attention to these matters at the end of 1909, hoping that many a working member of a committee may make himself familiar with the principles which have governed the proceedings of the committee of each institution with which he may be connected, and that he may resolve to use his influence to get these questions of pensions and holidays placed upon an adequate basis during the early part of 1910.

### NURSES AS SURGEONS.

Our Letter-Box brings us from time to time some extraordinary questions. Taken as they stand they at times appear ridiculous on the face of them. Many years' experience has taught us, however, that questions, like smoke, indicate a point of danger from fire, or worse. Here is one such question: "Is it desirable that nurses should learn and practise skin-grafting?" "Most certainly not," rises promptly to the lips; but what does the question mean at the bottom? With the object of learning more about it, we sent this question round to some of the chief medical superintendents, matrons, and members of the profession. In the result we find, to our astonishment, that at one or more of the great Poor Law Hospitals members of the medical profession have shown the nurses how to graft, and have allowed them to do it under their supervision. Other members of the same staff have asked the nurses to do it themselves, after they have been shown how to do it, despite the disapproval of the lady superintendent in charge. It will be seen, then, that the origin of this question and any abuse which it may represent is directly traceable to an indiscretion, or, as we hold, a dereliction of duty on the part of members of the medical profession connected with Poor Law Infirmaries, at any rate. The leaders of the nursing profession are unanimously opposed to this practice, holding properly that such an operation is entirely outside the province of a nurse. They maintain, further, that even where a practitioner may teach a nurse to graft in a case under his care, the practitioner is clearly wrong, for such teaching is not only undesirable, but might prove dangerous in practice. Skin-grafting is an operation which requires surgical skill and experience, and in our opinion should never be performed by a nurse. We should be glad to have the experience of practitioners and nurses, for it is a matter which concerns very closely the interests of both professions, and should not remain an open question.

### MEN AND MEASURES.

A contemporary humorous writer of some prominence—Mr. Barry Pain, to be specific—has remarked that to one so constantly engaged in criticism as the writer of leading articles to find himself the subject of it must be as good as a week at the seaside. Whether favourable or adverse comment provided the better tonic we are left in doubt: it is in the hope that the former is the case that attention is directed to a recent "leader" in the *Times* on the subject of Mr. Beit's Medical Research Fellowships. Stress is rightly laid on the importance of getting hold of the right men. "The academic qualification . . . is somewhat rigid. The true researcher is born not made and you never can tell where he may be found." As an instance, Sir Francis Galton's study of very eminent men of science, impartially selected, may be recalled. They were found to be not at all remarkable for academic distinction. Again—"Though modern research has become so elaborate that very costly appliances are in some fields quite indispensable, there is a tendency . . . to expect them to turn out results by an automatic process." Certainly the recent course of pathology has been more influenced by the private endeavour of one man than by all the labours at the Lister Institute, admirably equipped and devotedly served as that fine institution has been.

### THE DRUG MARKET.

The drug and chemical markets are now very quiet and business is not likely to be active until the second or third week in the New Year. Few price alterations of any importance have taken place and there are no indications which point with any degree of definiteness to probable alterations in the prices of drugs in the near future. Practitioners who use methylated spirit in any appreciable quantity should wait until January 1 before making purchases, as prices will then be reduced by 2d. per gallon.

